# Hot-Pressed Reinforced Photocatalyzed TiO_2_/Chitosan/SiO_2_ Nanofibers

**DOI:** 10.3390/ma18214828

**Published:** 2025-10-22

**Authors:** Jingwen Wang, Zunzhi Liu, Jingmei Zhang, Yang Liu, Chunjing Hou, Hui Cheng, Yaru Wang, Xiang Liu

**Affiliations:** 1 Xinjiang Key Laboratory of New Energy and Energy Storage Technology, Xinjiang Institute of Technology, Aksu 843100, China; 2023211@xjit.edu.cn (J.W.); 2019153@xjit.edu.cn (J.Z.); 18010615631@163.com (Y.L.); houchunjingjh@163.com (C.H.); hui_cheng@xjit.edu.cn (H.C.); wangyr@xjit.edu.cn (Y.W.); 2 Xinjiang Key Laboratory of Intelligent and Green Textile, College of Textiles and Clothing, Xinjiang University, Urumqi 830046, China

**Keywords:** electrospinning, photocatalysis, surface modification, titanium dioxide

## Abstract

This study introduces a novel fabrication method for high-strength, self-cleaning photocatalytic membranes through the integration of hot-pressing and TiO_2_/chitosan/SiO_2_ nanofibers. The innovation of this research lies in the hot-pressing technique, which significantly enhances the mechanical properties and photocatalytic efficiency by improving the adhesion, dispersion, and uniformity of the TiO_2_/chitosan coating on SiO_2_ nanofibers. SiO_2_ nanofibers with an initial diameter of 0.79 ± 0.29 μm were coated and hot-pressed, resulting in a final diameter of 1.07 ± 0.57 μm, which corresponds to an approximate increase of 35.4%. In addition, the 1 wt% TiO_2_-CTS sample showed the highest adhesion and surface energy, with values of 0.1 nN/nm^2^, indicating the closest intermolecular binding. 3 wt% SiO_2_-CTS exhibits a maximum hardness of 5.23 Pa. The 3 wt% TiO_2_-chitosan coating demonstrated outstanding mechanical performance, achieving a fracture stress of 0.53 MPa, approximately five times that of the untreated SiO_2_ nanofibers, a Young’s modulus of 0.63 MPa, and a toughness to triple of 0.27 MJ/m^3^—representing substantial improvements over uncoated membranes. Photocatalytic efficiency was significantly enhanced, with grayscale values increasing approximately 36% UV light exposure, indicating the superior degradation of pollutants.

## 1. Introduction

With the acceleration of industrialization and urbanization, environmental pollution, particularly air and water pollution, has become increasingly prominent [1,2,3,4]. Traditional cleaning methods are often inefficient and costly, leading to the exploration of new cleaning technologies [5]. Self-cleaning technology, as an emerging environmental protection technology, aims to reduce pollutant adhesion and improve material lifespan through special surface structures or functional coatings [6,7,8]. It has been widely applied in fields such as building materials [9,10], microfiltration membrane [11], and textiles [12]. However, due to the difficulty in separating the photocatalyst [13,14], there is an increased risk of the photocatalyst entering the environment, leading to secondary pollution [15].

To improve the recycling and reuse of photocatalysts, enhance their photocatalytic efficiency, and increase the mechanical properties of materials, several common methods have been developed [16,17,18]. These include immobilizing photocatalysts on substrates [19,20,21], combining them with magnetic nanoparticles to create magnetic porous membranes, and developing multilayer composite films with enhanced photocatalytic properties [22]. Additionally, optimizing photocatalytic performance and mechanical strength can be achieved through crosslinking and heat treatments.

At present, heterogeneous photocatalysts employed for dye degradation primarily encompass heterojunction photocatalysts, synergistic photocatalysts, composite oxides, and emerging visible-light-responsive materials such as CuO, BiVO_4_, ZnS, ZnO, AgBr and NiO. Among these materials, ZnO is widely employed as a photocatalyst owing to its wide band gap (Eg = 3.37 eV), high exciton binding energy (60 meV), and large specific surface area. Furthermore, the p-type semiconductor NiO has emerged as a research hotspot in photocatalysis due to its broad band gap range (3.2–4.0 eV), high photocatalytic activity, and strong capability in degrading organic pollutants [23,24,25,26,27]. A notable study by Tseng et al. explored the biomimetic preparation of hierarchical chitosan film surfaces using natural leaf structures as a template through nano-casting technology. In this study, photoactive TiO_2_ particles were generated on the chitosan film using the sol–gel method. The resulting structure demonstrated enhanced hydrophobicity, thermal stability, and CO_2_ adsorption capacity. Under UVA irradiation, the film could convert CO_2_ into carbon monoxide and trace amounts of methanol. These findings underscore the potential of integrating biomimetic designs with advanced material processing techniques to significantly enhance the functionality and durability of photocatalytic materials [28]. Cheng et al. developed a magnetic photocatalyst, ZnFe-BC, using a one-pot microwave heating method for the efficient removal of Rhodamine B (RB) and Malachite Green (MG) from wastewater. The study demonstrated that the photocatalyst exhibited significant adsorption capacities, achieving 334.89 mg/g for Rhodamine B and 576.73 mg/g for Malachite Green. These results highlight the potential of ZnFe-BC as an effective material for wastewater treatment applications [29]. Li et al. developed a novel composite membrane by incorporating CdS quantum dots into covalent triazine framework nanosheets. This innovative membrane demonstrated exceptional performance, achieving a high-water flux exceeding 170 L m^−2^·h^−1^ at 0.1 MPa and a dye retention rate greater than 94%. These findings suggest that the composite membrane holds significant potential for applications in advanced filtration and separation processes [30]. However, traditional methods face several challenges, including a reduction in the active sites of the photocatalyst, decreased photocatalytic efficiency due to the incorporation of magnetic materials, and the complexity of the preparation processes.

To address the limitations of traditional methods in enhancing the photocatalytic and mechanical properties of materials, this experiment proposes the dispersion of TiO_2_ onto chitosan, followed by coating the mixture onto a SiO_2_ nanofiber membrane. Chitosan is a linear polymer composed of glucosamine and N-acetylglucosamine monomers linked by β (1–4) glycosidic bonds. The presence of numerous amino and hydroxyl groups in its structure imparts considerable hydrophilicity. However, due to its high molecular weight, chitosan exhibits limited solubility despite strong film-forming capability. Furthermore, the abundant hydroxyl groups can form hydrogen bonds with water molecules, contributing to their excellent adhesive properties [31,32,33]. The process will be further optimized through hot pressing, which is intended to enhance the adhesion between the coating and the substrate, improve coating uniformity, and strengthen the mechanical properties of the membrane. Chitosan, rich in amino and hydroxyl groups [34], is known for its ability to complex with metal ions [35], thereby preventing agglomeration [36,37]. These functional groups can also form hydrogen bonds or other weak interactions with reactant molecules [38], facilitating their reaction with the catalyst’s active sites [39]. Additionally, chitosan offers excellent biocompatibility and film-forming properties, promoting better dispersion of metal ions on the support. The amino groups in chitosan exhibit electron-donating properties, which can inhibit electron-hole recombination, effectively extending the lifetime of photogenerated electrons. TiO_2_, a widely used photocatalyst with a bandgap of approximately 3.2 eV [40], enables the separation of photogenerated electrons and holes under UV light [41], leading to efficient photocatalytic performance [42]. In this experiment, a TiO_2_/chitosan (CTS) layer will be coated onto electrospun silica (SiO_2_) nanofiber membranes. The integration of chitosan with TiO_2_ is expected to enhance the mechanical strength and adhesion of the TiO_2_ coating, while also providing additional co-catalytic functions through the capture of photogenerated electrons by amino groups, thereby improving photocatalytic efficiency.

Organic dyes pose a serious threat to the ecosystem due to their mutagenic and cytotoxic properties. Therefore, photocatalytic dye degradation has significant environmental significance. Some dyes are regarded as potential carcinogens or mutagens, including azo, anthraquinone, sulfur, indigo, nitro, and nitrosyl dyes. Xylene orange is considered one of the main pollutants due to its biphenyl structure and stable chemical properties. Because titanium dioxide nanoparticles have strong surface reactivity, they are often used in dye degradation [43]. However, relevant studies indicate that nano-TiO_2_ induces cellular structural and oxidative damage in aquatic algae and plant organisms. It can either aggregate and exert physical pressure leading to cell wall deformation or enter cells via diffusion, thereby causing internal structural disruption. Once inside the cells, nano-titanium dioxide promotes the generation of reactive oxygen species, which ultimately leads to algal cell death. This toxicological mechanism limits the large-scale application of titanium dioxide in water treatment processes [44,45,46,47,48]. Furthermore, the application of high temperatures will enhance the dispersion and adhesion of the photocatalyst [49,50], further optimizing the material’s xylene orange photocatalytic performance. Hot pressing will enhance the formation of strong hydrogen bonds between the hydroxyl groups (-OH) in chitosan molecules and water molecules [51,52,53,54], potentially forming van der Waals forces or strong hydrogen bonds [55,56,57], thereby increasing surface energy and improving the overall mechanical properties of the material [58,59,60].

At present, heterogeneous photocatalysts employed for dye degradation primarily encompass heterojunction photocatalysts, synergistic photocatalysts, composite oxides, and emerging visible-light-responsive materials such as CuO, BiVO_4_, ZnS, ZnO, AgBr and NiO. Among these materials, ZnO is widely employed as a photocatalyst owing to its wide band gap (Eg = 3.37 eV), high exciton binding energy (60 meV), and large specific surface area. Furthermore, the p-type semiconductor NiO has emerged as a research hotspot in photocatalysis due to its broad band gap range (3.2–4.0 eV), high photocatalytic activity, and strong capability in degrading organic pollutants. A notable study by Tseng et al. explored the biomimetic preparation of hierarchical chitosan film surfaces using natural leaf structures as a template through nano-casting technology. In this study, photoactive TiO_2_ particles were generated on the chitosan film using the sol–gel method. The resulting structure demonstrated enhanced hydrophobicity, thermal stability, and CO_2_ adsorption capacity. Under UVA irradiation, the film could convert CO_2_ into carbon monoxide and trace amounts of methanol. These findings underscore the potential of integrating biomimetic designs with advanced material processing techniques to significantly enhance the functionality and durability of photocatalytic materials [23]. Cheng et al. developed a magnetic photocatalyst, ZnFe-BC, using a one-pot microwave heating method for the efficient removal of Rhodamine B (RB) and Malachite Green (MG) from wastewater. The study demonstrated that the photocatalyst exhibited significant adsorption capacities, achieving 334.89 mg/g for Rhodamine B and 576.73 mg/g for Malachite Green. These results highlight the potential of ZnFe-BC as an effective material for wastewater treatment applications [24]. Li et al. developed a novel composite membrane by incorporating CdS quantum dots into covalent triazine framework nanosheets. This innovative membrane demonstrated exceptional performance, achieving a high-water flux exceeding 170 L m^−2^·h^−1^ at 0.1 MPa and a dye retention rate greater than 94%. These findings suggest that the composite membrane holds significant potential for applications in advanced filtration and separation processes [25]. However, traditional methods face several challenges, including a reduction in the active sites of the photocatalyst, decreased photocatalytic efficiency due to the incorporation of magnetic materials, and the complexity of the preparation processes.

To address the limitations of traditional methods in enhancing the photocatalytic and mechanical properties of materials, this experiment proposes the dispersion of TiO_2_ onto chitosan, followed by coating the mixture onto a SiO_2_ nanofiber membrane. The process will be further optimized through hot pressing, which is intended to enhance the adhesion between the coating and the substrate, improve coating uniformity, and strengthen the mechanical properties of the membrane. Chitosan, rich in amino and hydroxyl groups [26], is known for its ability to complex with metal ions [27], thereby preventing agglomeration [28,29]. These functional groups can also form hydrogen bonds or other weak interactions with reactant molecules [30], facilitating their reaction with the catalyst’s active sites [31]. Additionally, chitosan offers excellent biocompatibility and film-forming properties, promoting better dispersion of metal ions on the support. The amino groups in chitosan exhibit electron-donating properties, which can inhibit electron-hole recombination, effectively extending the lifetime of photogenerated electrons. TiO_2_, a widely used photocatalyst with a bandgap of approximately 3.2 eV [32], enables the separation of photogenerated electrons and holes under UV light [33], leading to efficient photocatalytic performance [34]. In this experiment, a TiO_2_/chitosan (CTS) layer will be coated onto electrospun silica (SiO_2_) nanofiber membranes. The integration of chitosan with TiO_2_ is expected to enhance the mechanical strength and adhesion of the TiO_2_ coating, while also providing additional co-catalytic functions through the capture of photogenerated electrons by amino groups, thereby improving photocatalytic efficiency.

Furthermore, the application of high temperatures will enhance the dispersion and adhesion of the photocatalyst [35,36], further optimizing the material’s photocatalytic performance. Hot pressing will enhance the formation of strong hydrogen bonds between the hydroxyl groups (-OH) in chitosan molecules and water molecules [37,38,39,40], potentially forming van der Waals forces or strong hydrogen bonds [41,42,43], thereby increasing surface energy and improving the overall mechanical properties of the material [44,45,46].

## 2. Materials and Methods

### 2.1. Materials

The following materials were used: tetraethyl orthosilicate (99.99%, Aladdin, Shanghai, China), ethanol (≥99.5%, Sigma-Aldrich, Taufkirchen bei München, Germany), hydrochloric acid (≥37%, Aladdin, Shanghai, China), deionized water, chitosan (>400 mPa·s, Aladdin, China), glacial acetic acid (≥99.7%, Sigma-Aldrich, Taufkirchen bei München, Germany), and TiO_2_ nanoparticles (99.8%, nano-grade, Macklin, Shanghai, China), Orange II (Thermo Fisher Technology (China) Co., Ltd., Shanghai, China).

### 2.2. Preparation of Silicon Dioxide Nanofiber Membrane

**Figure 1:**
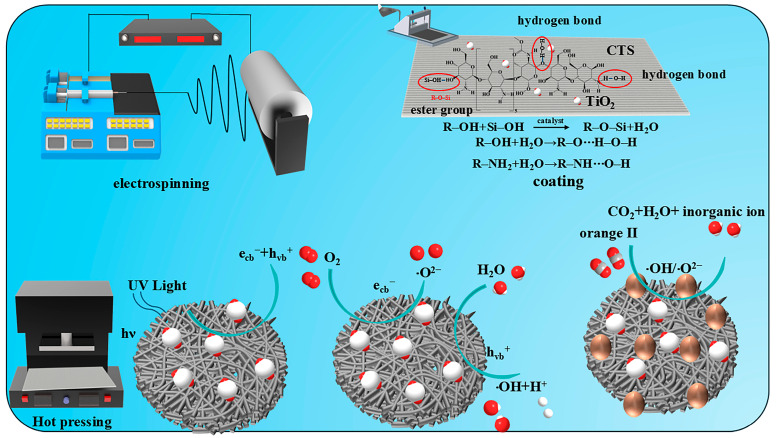
TiO_2_/chitosan/SiO_2_ nanofibers preparation process.

**Figure 2:**
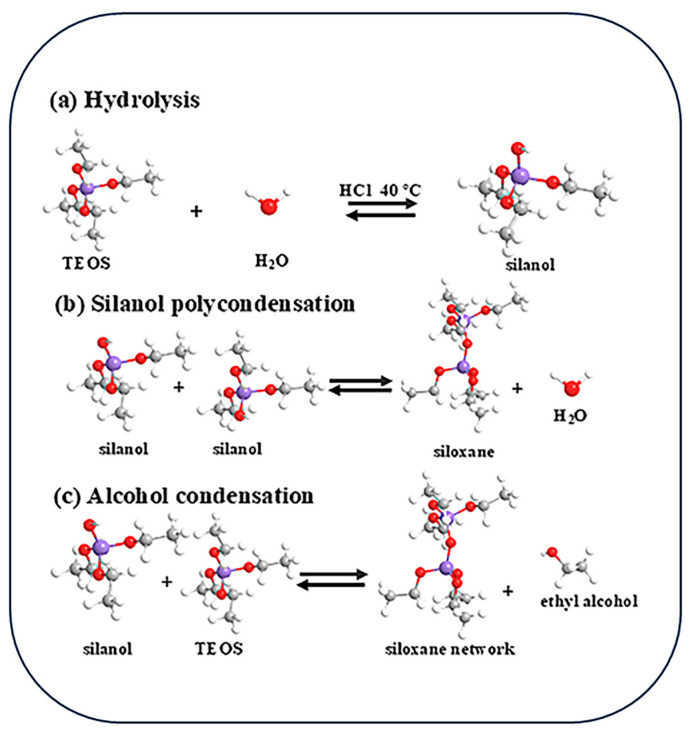
The reaction process of spinning solution.

Figure 1 exhibits preparation process of TiO_2_/chitosan/SiO_2_ nanofibers. The prepared fiber membrane was subjected to coating treatment. Under the combined effect of ester groups and hydrogen bonds, titanium dioxide was successfully loaded onto the surface of the silica fiber membrane. Subsequently, through hot-pressing treatment, the hydrogen bond combination between the fiber matrix and the coating was further strengthened. The spinning solution (Figure 2) is composed of 63 wt% TEOS, 8.9 wt% HCl (as a catalyst), and 26.3 wt% ethanol, all dissolved in ultrapure water to prepare a 30 mL solution. This solution is heated and stirred at 300 rpm and 80 °C, 30 min. The reaction process of spinning solution is shown in Figure 2. Electrospinning is conducted using a high-voltage power supply with a range of 16–20 kV. A 22-gauge needle is employed for spinning, with the spinning distance set at 15 cm. The spinning solution is fed to the needle at a rate of 0.5 mL/h using a syringe pump. The ambient temperature is maintained at 25 °C with a relative humidity of approximately 30%. Nanofibers are collected on a flat collector covered with aluminum foil. The spinning process lasts for 1 h, followed by natural drying of the film for 24 h, and subsequent drying in an oven at 60 °C for 12 h to obtain SiO_2_ nanofiber membranes.

### 2.3. Coating of SiO_2_ Nanofiber Membrane with TiO_2_/Chitosan Solution

1% (*w*/*v*) chitosan powder was added slowly to a container containing 2% (*w*/*v*) acetic acid. 1 wt%, 3 wt%, and 5 wt% TiO_2_ nanoparticles were dispersed in deionized water, then diluted with ultrapure water and adjusted the volume to 50 mL in a volumetric flask. Stir the mixture with a magnetic stirrer at room temperature for 4 h. The solution is then coated onto the surface of a SiO_2_ nanofiber membrane (5 cm × 5 cm) at room temperature using adjustable coating equipment, achieving a coating width of 30 mm ± 0.032 mm. After coating, the membrane is naturally air-dried for 12 h, followed by drying in a vacuum oven at 60 °C for 30 min to obtain a SiO_2_ nanofiber membrane with a TiO_2_/CTS coating.

### 2.4. Characterization of TiO_2_/Chitosan/SiO_2_ Nanofibers

#### 2.4.1. Scanning Electron Microscopy (SEM)

The morphology of the nanofibers was observed using a Supra 55 field emission scanning electron microscope (SEM) manufactured by Zeiss, Jena, Germany. Prior to testing, the sample surface was coated with a layer of platinum to enhance conductivity. The testing voltage was set at 10 kV.

#### 2.4.2. Nanoindentation

The indentation test was performed using a 0.5 μm Berkovich indenter at a constant strain rate of 0.1 s^−1^. The experiment began when thermal drift was below 0.5 nm/s. The nanoindentation test comprised both loading and unloading phases. The maximum load was set at 2 mN, with loading and unloading rates of 0.4 N/s and durations of 10 s for each phase. The distance between indentations varied between 10 and 30 mm, depending on the indentation load, with a total of five measurement points.

#### 2.4.3. Fourier Transform Infrared Spectroscopy Characterization (FT-IR)

FT-IR characterization was performed using a Zeiss Supra 55 infrared spectrometers. The scanning range was set from 400 to 4000 cm^−1^, with a resolution of 2 cm^−1^. A specific amount of catalyst was ground with potassium bromide at a 1:100 ratio, and approximately 50 mg of the mixture was pressed into a transparent disk for testing.

#### 2.4.4. Photocatalytic Degradation Test

0.8 g of xylenol orange was weighed and transferred into a beaker. A small amount of ethanol was added to dissolve the compound, then the solution was quantitatively transferred into a 50 mL volumetric flask and diluted to volume with ethanol, ensuring complete dissolution through stirring. Adjustable coating equipment was used to uniformly coat TiO_2_/chitosan/SiO_2_ nanofiber membranes with xylenol orange solutions at concentrations of 1 wt%, 3 wt%, and 5 wt%. A 3 W laboratory-scale UV lamp emitting light at a wavelength of 365 nm was used for irradiation. The nanofiber membranes were irradiated at a fixed distance of 3 cm in a light-tight environment to maintain consistent and controlled experimental conditions. The degradation performance of the film toward the dye was monitored at 3 min, 5 min, 10 min, 15 min, and 20 min by photographic recording, with the camera positioned at a fixed distance of 10 cm from the sample surface. Grayscale analysis was performed using ImageJ (ImageJ 1.52a) to evaluate the photocatalytic performance and determine the optimal formulation.

#### 2.4.5. Tensile Property

Ten test specimens (60 × 10 mm) were preconditioned for 48 h in a controlled environment at a temperature of 23 ± 1 °C and a relative humidity of 32 ± 5%. Tensile tests were performed using a ZQ-990A electronic tensile testing machine (Dongguan ZhiQu Precision Instruments Co., Ltd., Dongguan, China), equipped with a 100 N load cell, an initial grip separation of 30 mm, and a crosshead speed of 8.5 mm/min, in accordance with the ASTM D882-12 standard [61].

#### 2.4.6. Atomic Force Microscopy (AFM)

The atomic force microscope (AFM) used in this study was the Bruker Multimode 8 (Billerica, MA, USA). Prior to conducting mechanical tests, the AFM cantilever was calibrated by determining its spring constant and the sensitivity of the photodiode detecting the cantilever’s deflection. A suitable probe tip was selected, ensuring the probe maintained physical contact with the sample surface during scanning. The scanning range was set to 10 × 10 μm, with a loading rate of 2 mm/min.

## 3. Results

### 3.1. Morphology of TiO_2_/CTS/SiO_2_ Nanofibers

**Figure 3:**
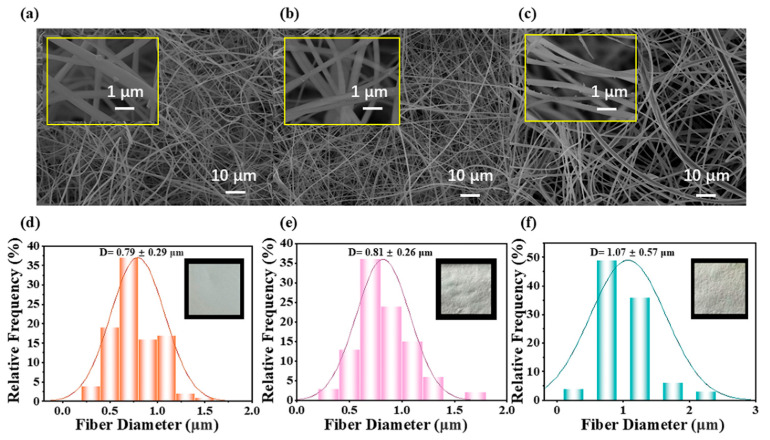
SEM of (**a**) SiO_2_ nanofibers, (**b**) SiO_2_ nanofibers after hot pressing, (**c**) SiO_2_ nanofibers coated with 5 wt% TiO_2_/CTS, Diameter distribution of (**d**) SiO_2_ nanofibers, (**e**) SiO_2_ nanofibers after hot pressing, and (**f**) SiO_2_ nanofibers coated with 5 wt% TiO_2_/CTS measured by Image J.

As depicted in Figure 3a, the SiO_2_ nanofiber membrane initially exhibits relatively small fiber diameters, averaging 0.79 ± 0.29 μm, as quantified in Figure 3d. These fibers are densely packed, forming a tightly intertwined three-dimensional network. Notably, the surfaces of the fibers are smooth and free from significant defects, indicating a high-quality fabrication process. Figure 3b reveals the morphological changes after the nanofiber membrane undergoes hot pressing. The fibers appear more orderly distributed, and there is no significant change in diameter compared with the original fibers, as shown in Figure 3e. This increase can be attributed to the softening fibers during hot pressing. Additionally, thermal expansion and fiber flow contribute to localized swelling, further influencing the fiber diameter. The consistency in the slight increase supports the conclusion that hot pressing enhances the structural integrity of the nanofiber membrane without compromising its overall morphology. After the SiO_2_ nanofiber membrane is coated with 5 wt% TiO_2_/CTS, as seen in Figure 3c, the fibers become more uniformly distributed, but their diameters exhibit greater variability. The average diameter increases significantly to 1.07 ± 0.57 μm, as highlighted in Figure 3f. This change is primarily due to the coating, which not only thickens the fibers but also introduces unevenness in their diameters. The increase in average diameter and variability underscores the impact of the TiO_2_/CTS coating on the nanofiber structure, suggesting a trade-off between coating benefits and morphological uniformity.

### 3.2. AFM Force Curves

**Figure 4:**
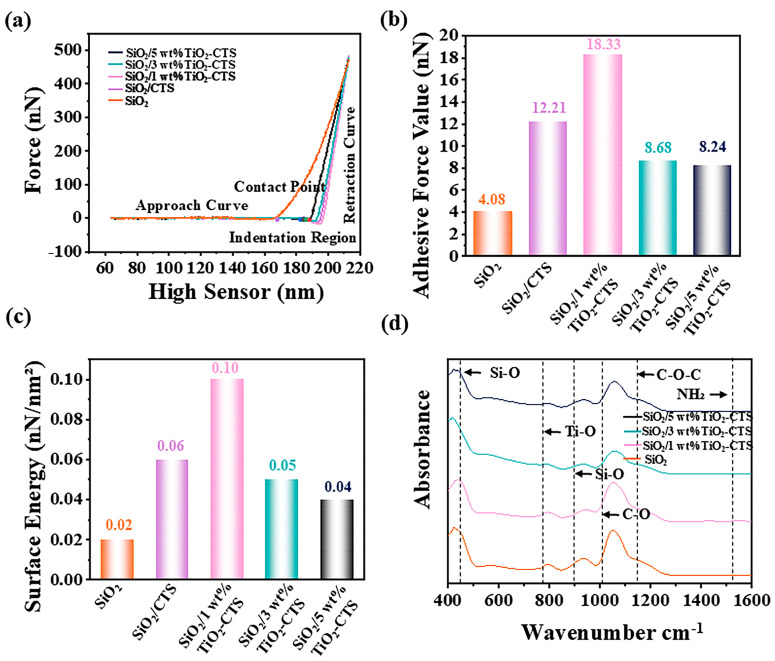
Sample’s (**a**) AFM Force Curves; (**b**) adhesive force; (**c**) surface energy of SiO_2_, SiO_2_-CTS, and SiO_2_ nanofibers coated with 1 wt%, 3 wt%, and 5 wt% TiO_2_/CTS; (**d**) FT-IR spectrum of SiO_2_ and SiO_2_ nanofibers coated with 1 wt%, 3 wt%, and 5 wt% TiO_2_/CTS.

As shown in Figure 4a, the SiO_2_ nanofiber membrane exhibits a lower maximum force and a smaller indentation region. This behavior indicates that the membrane has lower stiffness and is more susceptible to deformation, reflecting its relatively poor mechanical strength. The addition of CTS to the SiO_2_ nanofiber membrane significantly alters its mechanical properties. The SiO_2_-CTS membrane demonstrates a higher force value and a steeper curve in the indentation region, suggesting enhanced hardness and greater resistance to deformation due to the presence of CTS. The 1 wt% TiO_2_-CTS sample shows a relatively steep curve with a higher force value compared to the SiO_2_ nanofiber membrane, indicating that this concentration of TiO_2_-CTS imparts good mechanical strength and resistance to deformation. For the 3 wt% TiO_2_-CTS sample, the curve is slightly more gradual, with a corresponding slight decrease in force value. This implies that while the 3 wt% concentration still maintains commendable mechanical performance, there is a modest reduction in stiffness and deformation resistance. The 5 wt% TiO_2_-CTS sample presents the most gradual curve and the lowest force value among the samples. This suggests that the increased CTS content may soften the material or increase its plasticity, thereby reducing its hardness and resistance to deformation. Figure 4b displays the adhesive force values for different samples, revealing that the addition of CTS significantly increases the adhesive force on the SiO_2_ surface. This increase is likely due to the enhanced interaction between the CTS and the underlying SiO_2_ nanofibers, which improves surface adhesion. The 1 wt% TiO_2_-CTS sample exhibits the highest adhesive force, indicating that this concentration optimally enhances the surface interaction, leading to superior adhesion. As the TiO_2_-CTS concentration increases, the adhesive force gradually decreases. This reduction in adhesive force could be attributed to changes in the surface structure, possibly due to the higher content of TiO_2_ or CTS altering the distribution or effectiveness of surface-active sites. As shown in Figure 4c, the 1 wt% TiO_2_-CTS sample shows the largest surface energy of about 0.1 nN /nm^2^, indicating the tightest intermolecular binding.

### 3.3. FT-IR Characterization

Figure 4d presents the FT-IR spectra of different materials, providing insight into the chemical structure of the SiO_2_ nanofiber membranes. The vibrations observed at 450 cm^−1^ and 800 cm^−1^ are attributed to the symmetric stretching and bending vibrations of the Si-O bonds, confirming the presence of the Si-O network in the nanofibers. The broad band at 1200 cm^−1^ reflects the bending vibration of the C-O-C bond in an ester group, further verifying the presence of the ester group. Peaks in the 700 cm^−1^ correspond to the stretching vibrations of Ti-O and Ti-O-Ti bonds, characteristic of the TiO_2_ crystal structure. These peaks confirm the successful integration of TiO_2_ into the nanofiber matrix. As the material transitions from SiO_2_ nanofibers to 5 wt% TiO_2_-CTS nanofibers, the spectral peaks progressively broaden. This broadening is attributed to the presence of hydrogen bonds, which introduce additional complexity to the vibration modes, thereby leading to an increase in peak width. This bonding contributes to the enhanced stability and functionality of the coatings, suggesting that the combination of TiO_2_ and chitosan not only improves mechanical and surface properties but also reinforces the chemical integrity of the composite material.

### 3.4. Mechanical Characterization

**Figure 5:**
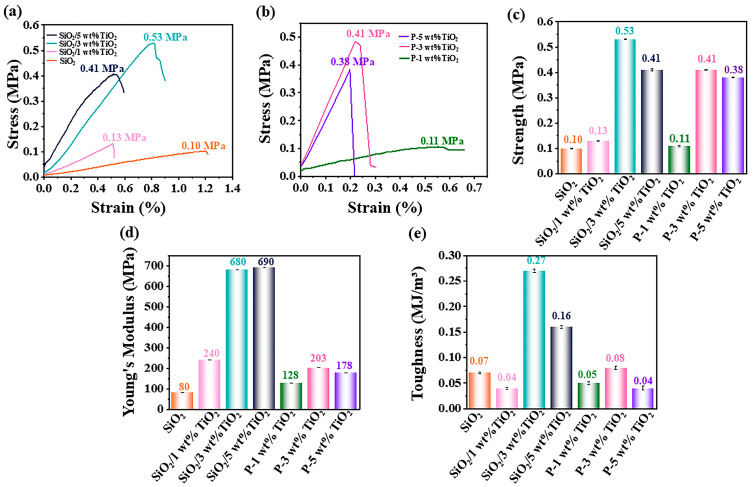
(**a**) Stress–strain curves of coated membrane, (**b**) stress–strain curves of photocatalytic membrane, (**c**) strength (**d**) Young’s modulus, and (**e**) toughness of nanofiber membrane.

As illustrated in Figure 5a, the stress–strain curves reveal significant changes in the mechanical performance of the nanofiber membranes. The uncoated SiO_2_ nanofiber membrane exhibits a maximum stress of approximately 0.1 MPa. Upon thermal pressing and subsequent coating with TiO_2_-CTS, the maximum stress values increase by 30% to 0.13 MPa, 0.53 MPa, and 0.41 MPa for membranes coated with 1 wt%, 3 wt%, and 5 wt% TiO_2_-CTS, respectively. This improvement indicates that the coating enhances the surface strength of the fibers, while thermal pressing improves fiber orientation and bonding, collectively leading to increased stress resistance. Notably, the 3 wt% TiO_2_-CTS coating achieves the highest stress value, suggesting an optimal balance of coating concentration and mechanical reinforcement. The results indicate that a coating concentration of 3 wt% is optimal for enhancing the mechanical properties of the material. When the coating loading increases to 5 wt%, strain strength decreases due to the agglomeration and lump formation of titanium dioxide during drying. This aggregation results in non-uniform coating distribution and inadequate dispersion within the matrix, which compromises stress transfer and ultimately reduces the material’s overall mechanical performance. As illustrated in Figure 5b, stress–strain performance tests were subsequently conducted on the films following photocatalytic treatment. After the experiment, the reductions in tensile stress for films coated with 1 wt%, 3 wt%, and 5 wt% titanium dioxides were measured as 0.02 MPa, 0.12 MPa, and 0.03 MPa, respectively. The observed stress reduction is attributed to the oxidative effects of free radicals generated during photocatalysis on the film material. Figure 5c focuses on the fracture stress of the nanofiber membranes, where the 3 wt% TiO_2_-CTS coating emerges as the most effective, achieving a fracture stress of 0.53 MPa. This finding underscores that 3 wt% is the optimal coating amount for maximizing the strength of the nanofiber membrane. The higher stress value at this concentration likely reflects an ideal distribution and interaction of the TiO_2_ nanoparticles with the fiber matrix. As shown in Figure 5d, the Young’s modulus of the SiO_2_ nanofiber membrane significantly increases after coating and thermal pressing. The uncoated membrane has a Young’s modulus of 0.08 MPa, which increases twice to 0.24 MPa, sevenfold to 0.63 MPa, and approximately octuple to 0.69 MPa for membranes coated with 1 wt%, 3 wt%, and 5 wt% TiO_2_-CTS, respectively. This substantial increase demonstrates the effectiveness of TiO_2_ as an inorganic filler in enhancing the stiffness of the nanofibers. The improved interface bonding strength between the coating and the nanofibers further contributes to the overall material stiffness. The Young’s modulus of the films coated with 1 wt%, 3 wt%, and 5 wt% titanium dioxide decreased by 112 MPa, 477 MPa, and 512 MPa, respectively, after the experiment. This reduction can be attributed to material degradation during the photocatalytic process, which led to the detachment of the surface coating.

Figure 5e evaluates the toughness of the nanofiber membranes, revealing a clear improvement with the addition of the TiO_2_-CTS coating and thermal pressing. The toughness of the uncoated SiO_2_ membrane is 0.07 MJ/m^3^. After coating and pressing, the toughness values for the membranes with 1 wt%, 3 wt%, and 5 wt% TiO_2_-CTS coatings are 0.04 MJ/m^3^, 0.27 MJ/m^3^, and 0.16 MJ/m^3^, respectively. The significant increase in toughness at 3 wt% TiO_2_-CTS again highlights this concentration as optimal, where the interaction between the coating and the nanofibers is most effective, thereby improving the material’s resistance to fracture under stress. The toughness of the films coated with 1 wt%, 3 wt%, and 5 wt% titanium dioxide was measured as 0.05 MJ/m^3^, 0.08 MJ/m^3^, and 0.04 MJ/m^3^, respectively, after the experiment. This reduction in toughness can be attributed to the introduction of surface defects following photocatalysis, which compromises the material’s ability to absorb energy before fracture.

### 3.5. Nanoindentation

**Figure 6:**
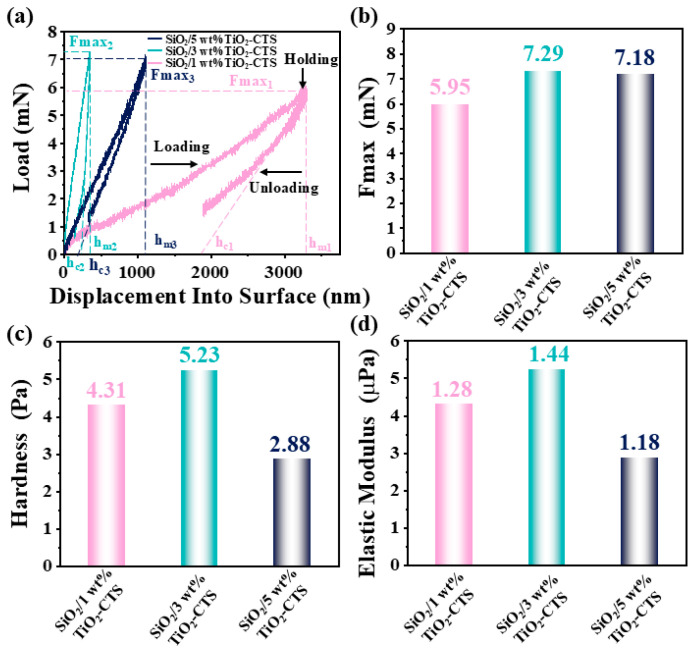
(**a**) Load–displacement curves; nanoindentation sampling location of (**b**) SiO_2_/1 wt% TiO_2_-CTS; (**c**) SiO_2_/3 wt% TiO_2_-CTS; (**d**) SiO_2_/5 wt% TiO_2_-CTS.

As illustrated in Figure 6a,b, the maximum load (Fmax) experienced by the SiO_2_ nanofiber membranes increases with the TiO_2_-CTS loading. Specifically, the Fmax values for the 1 wt%, 3 wt%, and 5 wt% loadings are 5.95 mN, 7.29 mN, and 7.18 mN, respectively. These results suggest that increasing the TiO_2_-CTS loading generally enhances the load-bearing capacity of the membranes, with the 3 wt% loading achieving the highest Fmax. This indicates that at 3 wt%, the material structure is optimized for handling maximum stress before deformation, likely due to the effective reinforcement provided by the TiO_2_-CTS coating. The hmax values are 3212.48 nm, 345.46 nm, and 1099.32 nm for the 1 wt%, 3 wt%, and 5 wt% loadings, respectively. The 1 wt% loading shows the largest displacement, indicating that this material is softer with a lower elastic modulus and poorer elastic recovery. This suggests that the nanofiber membrane with 1 wt% TiO_2_-CTS is more prone to deformation under load and less capable of returning to its original shape. In contrast, the 3 wt% loading exhibits the smallest displacement (345.46 nm), indicating the highest hardness and elastic modulus among the tested samples. This minimal displacement implies that the membrane is significantly more resistant to deformation and possesses superior elastic recovery, making it the most robust and resilient of the three compositions. The 5 wt% loading, with a displacement of 1099.32 nm, also demonstrates a relatively high hardness and elastic modulus, although slightly less than the 3 wt% sample. This suggests that while the 5 wt% loading improves the mechanical properties compared to the 1 wt% loading, it may introduce some degree of brittleness or less optimal distribution of the reinforcing TiO_2_-CTS particles, leading to a compromise between hardness and displacement. As exhibited in Figure 6c, the hardness (*H*) is calculated using the following formula [62]:

(1) $$H=\frac{Fmax}{A}$$

(2) $$A=24.5\times h_{c}^{2}$$

(3) $$h_{c}=h_{max}-\in \times \frac{Fmax}{S}$$

where *Fmax* is the maximum indentation load, *A* is the indented area, *h_max_* is the maximum displacement, ϵ is a constant related to the geometry of the indenter (ϵ ≈ 0.75 for a Berkovich indenter), and *S* is the initial slope of the unloading curve.

The calculated hardness values for SiO_2_ nanofibers with 1 wt%, 3 wt%, and 5 wt% loadings are 4.31 Pa, 5.23 Pa, and 2.88 Pa, respectively (Figure 6c). The elastic modulus (*Er*) is calculated using the following formula [63]:

(4) $$Er=\frac{S}{\sqrt[{2}]{A}}$$

where *S* is the slope of the unloading curve, and *A* is the contact area.

The calculated elastic modulus (*Er*) for SiO_2_ nanofibers with 1 wt%, 3 wt%, and 5 wt% loadings are 1.28 × 10^−6^ Pa, 1.44 × 10^−4^ Pa, and 1.18 × 10^−5^ Pa, respectively (Figure 6d).

### 3.6. Photocatalytic Property

Theoretically, the chitosan skeleton contains abundant amino groups (-NH_2_) and hydroxyl groups (-OH), which exhibit strong affinity for water molecules. Furthermore, the presence of lone electron pairs in -NH_2_ and -OH groups can create an electron-rich microenvironment, facilitating the separation of photogenerated holes and electrons in the valence band of TiO_2_. When titanium dioxide nanoparticles are irradiated with ultraviolet light at a wavelength of 365 nm, electrons in the valence band are excited and transition to the conduction band, leaving behind positively charged holes. These highly oxidative holes can oxidize water molecules to generate hydroxyl radicals (·OH), while the photoexcited electrons reduce molecular oxygen (O_2_) to form superoxide radical anions (·O_2_^−^). Subsequently, these reactive species attack the azo bond (-N=N-) in dye molecules, leading to their degradation into CO_2_, water, and inorganic ions.

**Figure 7:**
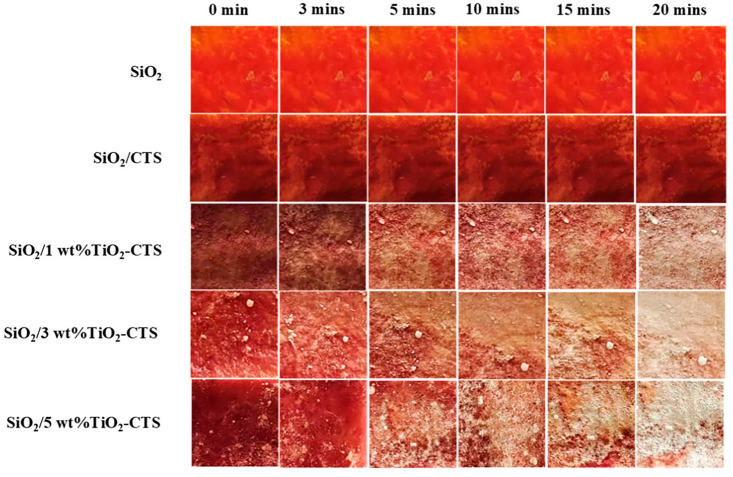
Degradation changes of SiO_2_, SiO_2_/CTS, and SiO_2_/1 wt%TiO_2_-CTS nanofiber films over different time intervals.

The pure SiO_2_ nanofiber film shows no significant color change even after 20 min of UV light exposure (Figure 7). This lack of color change can be attributed to the inherent absence of photocatalytic properties in SiO_2_ materials. SiO_2_ is known for its chemical stability and low reactivity under UV light, making it resistant to UV-induced degradation or color alteration. The SiO_2_/CTS film exhibits a darker color compared to the pure SiO_2_ film and shows no significant color change under UV exposure. The darker color can be explained by the addition of chitosan (CTS), which increases surface roughness and introduces more active sites on the material’s surface. These factors likely contribute to the aggregation of dyed molecules on the surface, resulting in a darker appearance. However, similar to SiO_2_, CTS itself does not possess photocatalytic properties, which explains the absence of color change under UV exposure. The 1 wt% TiO_2_/CTS sample shows noticeable color changes between 5 and 20 min of UV exposure. The presence of TiO_2_ introduces photocatalytic activity, enabling the degradation of surface-bound dye molecules, which is reflected in the observable color changes. The 1 wt% concentration is sufficient to initiate this photocatalytic process, though the changes are moderate. The 3 wt% TiO_2_/CTS sample exhibits more pronounced color changes under the same UV exposure conditions. Increasing the concentration of TiO_2_ enhances the photocatalytic activity, leading to more efficient degradation of dye molecules. The more pronounced color changes indicate that a higher concentration of TiO_2_ results in more active photocatalytic sites, accelerating the degradation process.

**Figure 8:**
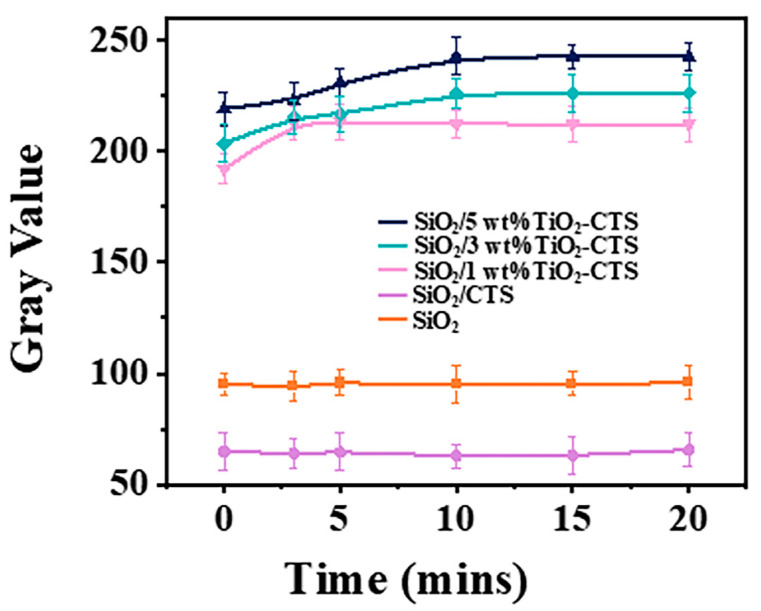
Gray value of nanofiber films over different time intervals.

To further assess the degradation intensity of SiO_2_ nanofiber films with different concentrations (Figure 8), the experiment utilized Image J to statistically analyze the grayscale values, with the following results: for pure SiO_2_ nanofibers, the grayscale values remained relatively stable, consistently around 65, indicating the lack of photocatalytic activity in SiO_2_ nanofibers. SiO_2_/CTS showed stable grayscale values with no significant changes, slightly higher than pure SiO_2_, indicating a slightly darker color, but with no change under light exposure, suggesting that the addition of CTS did not confer photocatalytic properties to the material. Under a concentration of 1 wt% TiO_2_-CTS, the grayscale values started at 191.9 and gradually increased to 213.1 within 5 min, remaining relatively stable, with an increase of 21.2, due to the photocatalytic action of TiO_2_. At a 3 wt% TiO_2_-CTS concentration, the trend of rising grayscale values was more pronounced, starting at 203.5 and reaching 225.9 within 10 min, relatively stable thereafter, with an increase of 22.4, indicating that the increase in TiO_2_ content enhanced the photocatalytic effect. At a 5 wt% TiO_2_-CTS concentration, the highest grayscale values were observed, starting at 218.9 and reaching 242.4 within 10 min, remaining relatively stable, with an increase of 23.5, suggesting that higher concentrations further enhanced the photocatalytic degradation performance, potentially leading to faster and more thorough dye degradation. This improvement can be attributed to the high coating loading, which provides a greater number of active sites for catalyst, thereby enhancing the photocatalytic performance of the film. The data indicate that the addition of TiO_2_ significantly affects the photo responsive behavior of SiO_2_/CTS composite materials under UV light exposure. As the TiO_2_ concentration increases, the photocatalytic degradation performance of the material improves, resulting in rapid degradation of the dye components.

## 4. Conclusions

This study focuses on developing high-strength photocatalytic membranes by hot-pressing TiO_2_/CTS/SiO_2_ nanofibers. Initially, SiO_2_ nanofibers with a diameter of 0.79 ± 0.29 μm increased to 1.07 ± 0.57 μm following the application of TiO_2_/CTS coating and hot pressing. Among the various coatings, the 3 wt% TiO_2_-CTS coating exhibited the best mechanical properties, achieving a fracture stress of 0.53 MPa, a Young’s modulus of 0.63 MPa, and a toughness of 0.27 MJ/m^3^. Additionally, photocatalytic efficiency was enhanced, with grayscale values increasing up to 23.5 under UV light exposure. These membranes, which combine mechanical strength and photocatalytic activity, are well-suited for applications requiring durability and self-cleaning capabilities, such as in construction and textiles.

## Data Availability

The original contributions presented in this study are included in the article. Further inquiries can be directed to the corresponding author.
